# Qinghao-Biejia Herb Pair Alleviates Pristane-Induced Lupus-Like Disease and Associated Renal and Aortic Lesions in ApoE^−/−^ Mice

**DOI:** 10.3389/fphar.2022.897669

**Published:** 2022-04-29

**Authors:** Jiaze Hong, Miao Zhang, Yuanfang He, Yi Jin, Qiaoqi He, Yi Zhang, Xiaowei Shi, Weiyu Tian, Chengping Wen, Juan Chen

**Affiliations:** ^1^ The Second Clinical Medical College, Zhejiang Chinese Medical University, Hangzhou, China; ^2^ College of Basic Medical Science, Zhejiang Chinese Medical University, Hangzhou, China

**Keywords:** Qinghao-Biejia herb pair, systemic lupus erythematosus, atherosclerosis, inflammatory activity, HMGB1/TLR4 signaling pathway, mice

## Abstract

**Backgroud:** Systemic lupus erythematosus (SLE) is a chronic autoimmune disease involving multiple systems with a high prevalence of nephritis and atherosclerosis. Jieduquyuziyin prescription is a famous prescription with immune modulating and inflammation controlling effects, which is efficacious in the treatment of SLE. The most critical herbs in this prescription are Qinghao and Biejia. The aim of this study was to evaluate the therapeutic effect of Qinghao-Biejia herb hair (QB) on mice with SLE combined with atherosclerosis.

**Materials and Methods:** The effect of QB (identification using UPLC-TOF-MS) was assessed in female ApoE^−/−^ mice intraperitoneally injected with 0.5 ml of pristane. Serum autoantibodies and lipid metabolic parameters were tested every 4 weeks, and spleen index, serum inflammatory biomarkers, renal injury, and aortic injury were observed after 16 weeks. The expression of signaling pathway in kidney tissues was observed by RT-qPCR and Western blot.

**Results:** The mice of QB-treated group exhibited a significant reduced serum autoantibodies level, urine protein, and renal immune complex deposition. QB treatment reduced the levels of inflammatory cytokines and improved the renal pathological changes. In addition, there was a reduction in aortic atheromatous plaque and some improvement in dyslipidemia. Moreover, QB suppressed the expression of HMGB1, TLR4, and MyD88 to some extent.

**Conclusion:** The present study implied that QB has clear efficacy for the treatment of SLE combined with atherosclerosis, and that inhibition of the HMGB1/TLR4 signaling pathway may be one of the therapeutic targets of QB for SLE combined with atherosclerosis.

## Introduction

Systemic lupus erythematosus (SLE) is a systemic autoimmune disease characterized by the formation of nuclear autoantigens (Soni et al., 2020), which can cause immune complex formation resulting in inflammation of multiple organs (Durcan et al., 2019). With the development of diagnosis and management, although survival in SLE has improved substantially in the last decades, various complications are still challenging problems in the treatment of SLE (Munguia-Realpozo et al., 2019; Tselios et al., 2019). The view that the bimodal pattern of excess mortality in SLE is generally accepted now, that the early peak being primarily the consequence of active lupus or increased infection risk and the late peak predominantly attributable to cardiovascular disease (CVD) (Øhlenschlaeger et al., 2004; Symmons and Gabriel, 2011; Ryu et al., 2018). The current treatments for SLE include corticosteroids, anti-malarial, and various immunosuppressive agents (Fava and Petri, 2019). Although these conventional treatments can alleviate certain symptoms and temporarily prevent disease progression, their therapeutic efficacy is limited by medication-related toxicity, and the inability to provide palliation of renal and CVD.

Current studies have showed that traditional Chinese medicine (TCM) treatment or combined treatment with chemical drugs can reduce the adverse events of drugs and the dosage of corticosteroids, improve the symptoms of SLE patients, enhance the body’s self-immune regulation ability, and consolidate the clinical efficacy (Ma et al., 2016; Chen et al., 2019). Jieduquyuziyin prescription is one of the well-known prescriptions for the treatment of SLE in contemporary China. A number of clinical studies have reported that Jieduquyuziyin prescription is reliably efficacious in the treatment of SLE (Wen et al., 2007; Yang et al., 2021). *Artemisia annua L.* (Compositae) (Sweet wormwood) (ancient name: Qinghao) and *Carapax Trionycis* (Trionychidae) (Turtle Shell) (ancient name: Biejia), the core components of this prescription, are two complementary traditional Chinese herbs that together form a herb pair. In our previous study, we found that Qinghao-Biejia herb pair (QB) could improve the pathological manifestations of lupus nephritis (LN). It can also exert therapeutic effects on SLE mice by regulating their lipid metabolism (Gan et al., 2015; Chen et al., 2016a). However, little is known about its exact mechanism and drug targets, thus there is a need to find new drug targets for SLE and co-morbid atherosclerosis disorders.

It is worth noting that disorders of inflammatory signaling is a common cause of SLE and atherosclerosis (Li et al., 2015; Ayoub et al., 2008). High mobility group box 1 (HMGB1) is an intranuclear protein that has been shown to play an important role in mediating inflammation when released from apoptotic or necrotic cells as a damage-associated molecular pattern (Liu et al., 2020). It is also an endogenous immune cofactor that estimates the severity and expression of SLE (Schaper et al., 2017; Tanaka et al., 2019). Toll-like receptor 4 (TLR4), a transmembrane non-catalytic protein expressed on the cell surface, is a downstream target of HMGB1 and is involved in the induction of the inflammatory program and in the progression of SLE and atherosclerosis (Michelsen et al., 2004; Hu et al., 2016). HMGB1 can activate and chemotacticize inflammatory cells, participate in TLR4 mediated immune responses, activate nuclear factor kappa-B (NF-κB) through Myeloid differentiation primary response 88 (MyD88), lead to the release of inflammatory factors, and thus promote the development and progression of SLE and atherosclerosis (Porto et al., 2006; Xiao et al., 2016). Furthermore, our preliminary results showed that QB could ameliorate renal lesions in lupus MRL/lpr mice by inhibiting the expression of TLR4. Based on these, we propose the hypothesis that QB can reduce the inflammatory response and delay disease progression in SLE combined with atherosclerosis by inhibiting the HMGB1/TLR4/MyD88 pathway.

This study aimed to investigate the potential protective effects of QB in SLE combined with atherosclerosis mice, i.e., improvement of renal and atherosclerotic lesions, reduction of immune complex deposition, inhibition of inflammatory responses, and modulation of lipid metabolism. We selected high-fat-fed pristane-induced ApoE^−/−^ mice to establish a suitable animal model of SLE combined with atherosclerosis. To our knowledge, there are no previous studies on the protective effects of QB in the *in vivo* treatment of SLE combined with atherosclerosis. In addition, another aim of this study was to explore the underlying molecular mechanisms of protection by QB.

## Materials and Methods

### Preparation of Qinghao-Biejia Decoction and Chemicals

*Artemisia annua L.* (Compositae) (Sweet wormwood), with the ancient name of Qinghao, is an annual, erect herb with a characteristically strong fragrance. The use part is the whole herb. *Carapax Trionycis* (Trionychidae) (Turtle Shell), with the ancient name of Biejia, is the tergum of the Trionyx sinensis Wiegmann. Raw herbs of Qinghao and Biejia were obtained from Zhejiang Chinese Medical University Medical Pieces Co., Ltd. (Hangzhou, China). Voucher specimens of the above two herbs were stored in the specific Herbarium room of Zhejiang Chinese Medical University. Decoction was prepared by the drug production room of Affiliated hospital, Zhejiang Chinese Medical University. The specific decoction methods were as follows. QB decoction (concentration: contains 0.42 g/ml of raw drug): pour a whole piece of Biejia (162 g) and its infusion solution (about 250–300 ml) into the tisanes casserole and decoct for 30 min, then add 22 g of Qinghao and its infusion solution and decoct for 10 min, filter the decoction through a strainer, and collect 150–200 ml filtrate; add 22 g of Qinghao and its infusion solution, and decoct it for another 10 min, pour out and collect 150–200 ml filtrate of the decoction, repeat the above steps once; concentrate the decoction to 270 ml by rotary evaporation and then dilute it to half of the original concentration. Qinghao decoction (concentration: contains 0.12 g/ml of raw drug) and Biejia decoction (concentration: contains 0.12 g/ml of raw drug): use a decoction method similar to the one described above. The concentrations of drugs are proportionally converted from the commonly used clinical dose. The final aqueous extract of QB was homogenized and stored in a sealed container at −20°C. Hydroxychloroquine (HCQ) sulfate tablets was purchased from Shanghai Shangyao Midwest Pharmaceutical Co., Ltd. (Shanghai, China) (China Food and Drug Administration Permission Number: H19990263).

### Ultrahigh-Performance Liquid Chromatography Time of Flight Mass Spectrometry for Identification and Content Determination

The main contents of QB decoction were analyzed by ultrahigh-performance liquid chromatography time of flight mass spectrometry (UPLC-TOF-MS) for quality control. The temperature was settled at 35°C and the chromatographic column used was a CORTECS UPLC T3 column (2.1 mm × 100 mm, 1.67 μm) (Waters, United States). The sample volume was 2 μl, and the flow rate was 1.0 ml/min. The mobile phases were a mixture of acetonitrile (A) and 0.1% acetic acid (B). The gradient elution program was used: 0–30.00 min, 10%–100% A; 30.01–31.00 min, 100%–10% A; 31.01–35.00 min, 10%–100% A.

The UPLC-TOF-MS used SYNAPT G2-Si ion mobility mass spectrometer (Waters, United States). The electrospray ionization (ESI) was used in positive and negative mode, and the mass-to-charge ratio (m/z) scan range was set at 50–1,000 Da. Mass spectrometry data were collected in centroid MS^E^ mode. The relative molecular mass accuracy was automatically calibrated using the tuned liquid transfer system from Waters. The instrument operation and data acquisition were controlled by the Waters MassLynx 4.1 system, and the compounds were identified according to the relative retention time and mass spectrometry information of each compound.

### Animals

Female ApoE^−/−^ mice with a C57BL/6 background (*n* = 60; 7 weeks old; weight, 18.0–20.4 g) were purchased from Beijing Weitong Lihua Laboratory Animal Technology Co., Ltd. (Beijing, China) and maintained in specific pathogen free (SPF) units of Barrier Animal Experimental Facility of Animal Experimental Research Center at Zhejiang Chinese Medical University (temperature: 23 ± 2°C; relative humidity: 60%–70%) with a 12 h light/dark cycle (light on at 7:00 a.m.) in conventional cages [certificate: SYXX (Zhe) 2018-0012]. The experimental mice were housed 5 per cage and were accessed to water and food freely. All female mice were given 1 week adaptive feeding before the experiment start. All animal experimental procedures conformed to the NIH Guidelines for the Care and Use of Laboratory Animals (Jones-Bolin, 2012).

### Experimental Design and Drug Treatments

After 1 week of adapted feeding, 60 female mice were randomly divided into the following six groups (10 in each group): ApoE^−/−^ group (control group), ApoE^−/−^ + Pristane group (model group), HCQ group, Qinghao-Biejia group (QB group), Qinghao group (Q group), and Biejia group (B group). Each group of ApoE^−/−^mice were fed with high-fat diet containing 0.5% cholesterol and 20% fat (Jiangsu Xietong Pharmaceutical and Bioengineering Co., Ltd., Nanjing, China) for consecutive 16 weeks (Zhou et al., 2019). The mice in model group, HCQ group, QB group, Q group, and B group (all five groups above were SLE mice) were given 0.5 ml of pristane by intraperitoneal injection respectively. Ones in control group were intraperitoneally injected with 0.5 ml of saline. Mice in SLE groups were confirmed by the measurement of autoantibodies 4 weeks after pristane injection: anti-double-stranded DNA antibodies (anti-dsDNA) and antinuclear antibodies (ANA) in serum expressed at levels significantly higher than the relative expression levels of control mice (*p* < 0.05) (Yan et al., 2020). After the mice models of SLE combined with atherosclerosis were established, HCQ group, QB group, Q group, and B group were given a gavage of HCQ, QB decoction, Qinghao decoction, and Biejia decoction (10 ml·kg^−1^·d^−1^) respectively. Control group and model group were given saline of the same volume with the drugs by gavage.

Supplementary Figure S1 describes the experimental protocol.

### Determination of Autoantibodies

The levels of anti-dsDNA and ANA in serum was determined by Enzyme-Linked Immunosorbent Assay (ELISA) kits (Alpha Diagnostic International, San Antonio, United States), according to the manufacturer’s instructions. Briefly, the serum was added to the 96-well plates and absorbance (OD value) was measured at a wavelength of 450 nm. A reference standard curve was prepared with mouse anti-dsDNA monoclonal antibody and ANA, and the concentrations were quantified by the standard curve.

### Measurement of Weight and Spleen Index

All mice were weighed at week 0, week 4, week 8, week 12, and week 16 after the start of the experimental procedure, and their weights were recorded. After administration, the mice were executed, and the spleen tissues were collected and weighed. Immediately afterwards, photographs were taken with a digital camera. Spleen index were calculated according to calculation formula. The formula of spleen index: spleen weight (g)/body weight (g) × 100%.

### Determination of Proteinuria

Urine samples were obtained by metabolic cages at week 8, week 12, and week 16 after the start of the experimental procedure, respectively. The collected urine samples were centrifuged at 3,000 r/min for 5 min to remove all the sediment, then the supernatant was collected and frozen at −20°C prior to use. Urine protein was measured by a Urine Protein Quantitative Test Kits (Jiancheng Bioengineering Institute, Nanjing, China). The data were expressed as millimole of albumin per liter urine.

### Measurement of Serum Lipid Metabolic Parameters

The blood samples were allowed to clot at 4°C and centrifuged at 3,000 r/min for 15 min, the serum samples were sterilized by microporous membrane filtration and stored at −20°C refrigerator until assayed. The blood samples were tested for the measurement of total cholesterol (TC), triglyceride (TG), low density lipoprotein cholesterol (LDL-C), and high density lipoprotein cholesterol (HDL-C) with commercial kits (Mecon Biotech Co., Ltd., Ningbo, China).

### Histopathological and Immunohistochemical Analysis

HE and EVG Staining: kidney and aortic samples from mice were preserved separately in frozen 4% paraformaldehyde, made into paraffin blocks, and then sectioned at a thickness of 4 μm. Next, dewaxing was performed with xylene for 10 min (x2), and then the samples were washed (x6) with a graded ethanol series (95%, 90%, 80%, 75%, and 70% dilution with distilled water). After washing, the kidney samples were sequentially stained with hematoxylin for 3 min and eosin in 95% ethanol solution for 3 min before rinsing with distilled water. The tissue was dehydrated with absolute ethanol and sealed with neutral resin. EVG staining was used to observe whether there was hyperplasia or fracture disintegration of elastic fibers within the aorta. Paraffin sections were removed for dewaxing and dehydration, followed by staining with Verhoeff solution (alcohol hematoxylin: ferric chloride: iodine solution = 5:2:2 preparation) for 30 min. The degree of background differentiation was controlled under the microscope with ferric chloride differentiation solution, and then the sections were re-stained with Van Gieson solution (saturated picric acid: acidic magenta = 9:1 preparation) for 3 min, and transparently sealed. Ultimately, images of the sample were collected by a light microscopic (Nikon, Japan) photographing.

Immunohistochemistry Staining: the density of C3 and IgG in the kidney was determined by IHC. Kidney tissue was paraffin-embedded and tissue sections (4 μm thick) were immunohistochemically stained according to standard procedures. Briefly, after incubation in 3% H_2_O_2_ for 10 min, microwave treatment for antigen retrieval and 5% BSA blocking, sections were incubated with rabbit anti-mouse C3 primary antibody (Abcam, #ab200999) and anti-mouse IgG primary antibody (Abcam, #ab190475) in a moisture box at 4°C overnight. The sections were then incubated with goat anti-rabbit IgG H&L secondary antibody (Abcam, #ab97051) for 30 min at room temperature, followed by color development using DAB and re-staining with hematoxylin. Sections were visualized with a light microscopic (Nikon, Japan), and sites with brownish-yellow particles precipitating were judged as positive.

### Inflammatory Cytokines Analysis

Total serum levels of interleukin-6 (IL-6), tumour necrosis factor alpha (TNF-α), interferon alpha (IFN-α), interferon gamma (IFN-γ), macrophage inflammatory protein-1 alpha (MIP-1α), and eotaxin were determined by Bio-Plex Pro Cytokine, Chemokine, and Growth Factor Assays (Bio-Rad, United States). IL-6, interleukin-1 beta (IL-1β), and TNF-α in kidney were measured with commercial ELISA kits (MultiSciences, Hangzhou, China). All assays were performed in strict accordance with the manufacturer’s protocols. All samples were taken in triplicate. The absorbance of each well of the 96-well plate was read at a wavelength of 450 nm with a microplate spectrophotometer (Nikon, Japan).

### Real-Time Quantitative Polymerase Chain Reaction

Total RNA was extracted from kidney and aorta tissue by RNAiso Plus reagent (TAKARA Bio Inc., Dalian, China), and then was transformed into cDNA using the TAKARA Reverse Transcription System Kit (Agbio Bio Inc., Changsha, China) according to the manufacturer’s instructions. The RT-qPCR was performed with Roche LightCycler 96 SW1.1 instrument (Roche, Basel, Switzerland) using the SYBR Green Premix Pro Taq HS qPCR Kit (Agbio Bio Inc., Changsha, China). The RT-qPCR conditions were as follows: 95°C for 30 s followed by 40 cycles of 95°C for 30 s, and 60°C for 30 s. All samples were analyzed in duplicate. The cycle number at which transcripts could be detected (Ct) was normalized as the cycle number of glyceraldehyde-3-phosphate dehydrogenase (GAPDH) gene detection, and referred to as ΔCt. The average of the values was calculated, and the quantification was analyzed by the 2^−ΔΔCt^ method. The RT-qPCR primer sequences (designed by TAKARA Bio Inc., Dalian, China) are shown in Table 1.

**TABLE 1:** Primer sequences required for RT-qPCR.

Gene	Forward sequence (5′ to 3′)	Reverse sequence (5′ to 3′)
HMGB1	AGA​TAT​GGC​AAA​GGC​TGA​CAA​GGC	GGG​CGG​TAC​TCA​GAA​CAG​AAC​AAG
TLR4	TCC​TGT​GGA​CAA​GGT​CAG​CAA​C	TTA​CAC​TCA​GAC​TCG​GCA​CTT​AGC​A
MyD88	AAG​ATG​ACC​CTG​GGA​GCC​CTA	CTC​AGG​CCA​GTC​ATC​ATT​GAA​CA
GADPH	GAACGGGAAGCTCACTGG	GCC​TGC​TTC​ACC​ACC​TTC​T

HMGB1, high mobility group box 1 protein; TLR4, toll like receptor 4; MyD88, myeloid differentiation primary response 88; GADPH, glyceraldehyde-3-phosphate dehydrogenas.

### Western Blot

The kidney tissue was homogenized in radio immunoprecipitation assay (RIPA) protein lysis buffer (Beyotime Biotechnology, Shanghai, China) using a ball mill (RETSCH, Haan, Germany) and the concentrations of the extracted proteins were measured using a BCA Protein Assay Kit (Biosharp, Hefei, China). Protein was separated by 10% SDS-PAGE gel (80 V, for 1 h) and transferred onto a nitrocellulose filter membrane (PALL, NY, United States) (300 mA, for 90 min). Thereafter, the membrane was blocked with 5% (weight in volume) skim milk solution in TBST buffer for 1 h at room temperature, and incubated overnight at 4°C with primary antibodies. Subsequently, membranes were washed and incubated with goat anti-rabbit IgG H&L secondary antibody at room temperature for 1 h. The primary antibodies used in this study were anti-HMGB1 (1:1000 dilution, Abcam, #ab18256), anti-TLR4 (1:1000 dilution, Thermo Fisher Scientific, #MA516216), and anti-MyD88 (1:1000 dilution, CST, #4283S). The images were quantified using ImageJ software and quantitated levels were normalized to their respective blotting from β-actin (1:2000 dilution, CST, #4970S). Three independent assays were performed.

### Statistical Analysis

The measurement data were expressed as mean ± SEM. Significant differences between the groups were determined by one-way analysis of variance (ANOVA) and Dunnett’s multiple comparison test, and *p* < 0.05 was considered statistically significant. Statistical analyses were performed using SPSS 26.0 software (IBM Corporation, Armonk, NY, United States). The images in this article were created by GraphPad Prism 8.0 (GraphPad Software Inc., La Jolla, CA, United States).

## Results

### Quantitative Analysis of the Chemical Constituents

UPLC-TOF-MS was applied for the rapid qualitative study of QB decoction. Finally, 38 and 58 chemical components were identified respectively in the negative and positive ion modes of QB decoction.74 compounds were identified by summing up the negative and positive modes and removing the common compounds (Supplementary Figure S2 and Supplementary Table S1).

### General Characteristics in Systemic Lupus Erythematosus Combined With Atherosclerosis Mice

**FIGURE 1:**
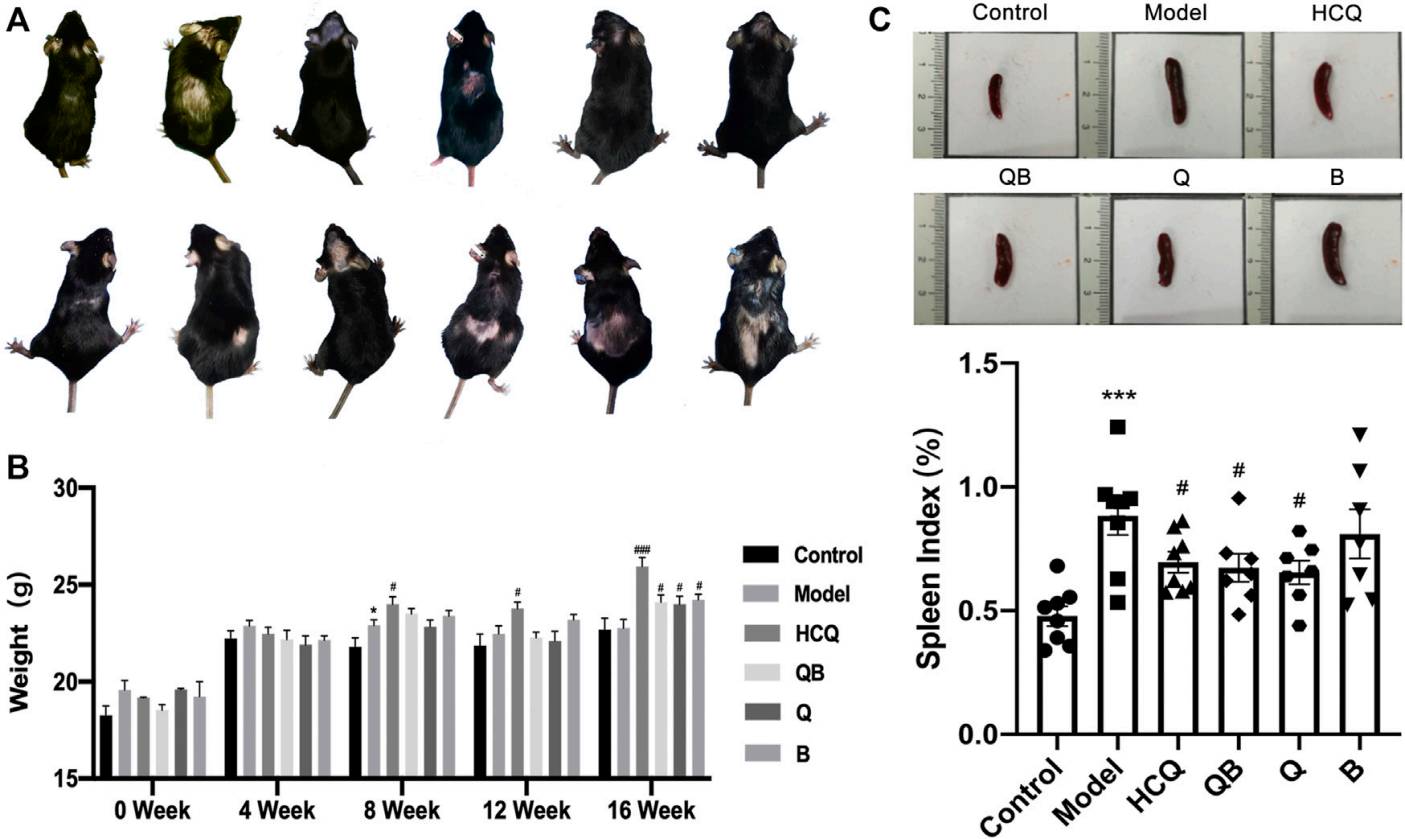
General characteristics in systemic lupus erythematosus (SLE) combined with atherosclerosis mice. **(A)** Hair condition of experimental mice. **(B)** The body weight of mice. **(C)** The spleen volume and spleen index of mice. The data are expressed as the mean ± SEM. Model group compared with the control group, *p < 0.05, ***p < 0.001; the remaining groups compared with the model group, ^#^ *p* < 0.05, ^###^ *p* < 0.001.

At 4 weeks after pristane injection, 12 of 50 ApoE^−/−^ C57BL/6 mice were observed to have varying degrees of dorsal hair loss (Figure 1A). During 16-week administration of drug, no animal deaths or signs of toxicity were observed in any group. During 0–4 weeks, mice in all groups had fair mental status, fair feeding status, and moderate spontaneous activity. From the fifth week onwards, there was some reduction in spontaneous activity in the model group of mice. After 16 weeks of drug intervention, the body weights of mice in the HCQ, QB, Q, and B groups were significantly higher than that of mice in the model group (Figure 1B).

The spleen is the largest immune organ in the body, and SLE patients usually develop splenomegaly due to immune dysregulation (Yan et al., 2020). We assessed the level of splenomegaly by testing the splenic index (organ weight/body weight × 100%), which is one of the important indicators of SLE progression (Watson et al., 1992) (Figure 1C). This study showed that mice in the model group had significantly more severe splenomegaly compared to mice in the control group. In contrast, mice in the QB, Q, and HCQ groups had significantly lower splenic indices, indicating that QB, Qinghao, and HCQ could effectively improve the symptoms of splenomegaly caused by SLE.

### Qinghao-Biejia can Reduce Autoantibody Production

**FIGURE 2:**
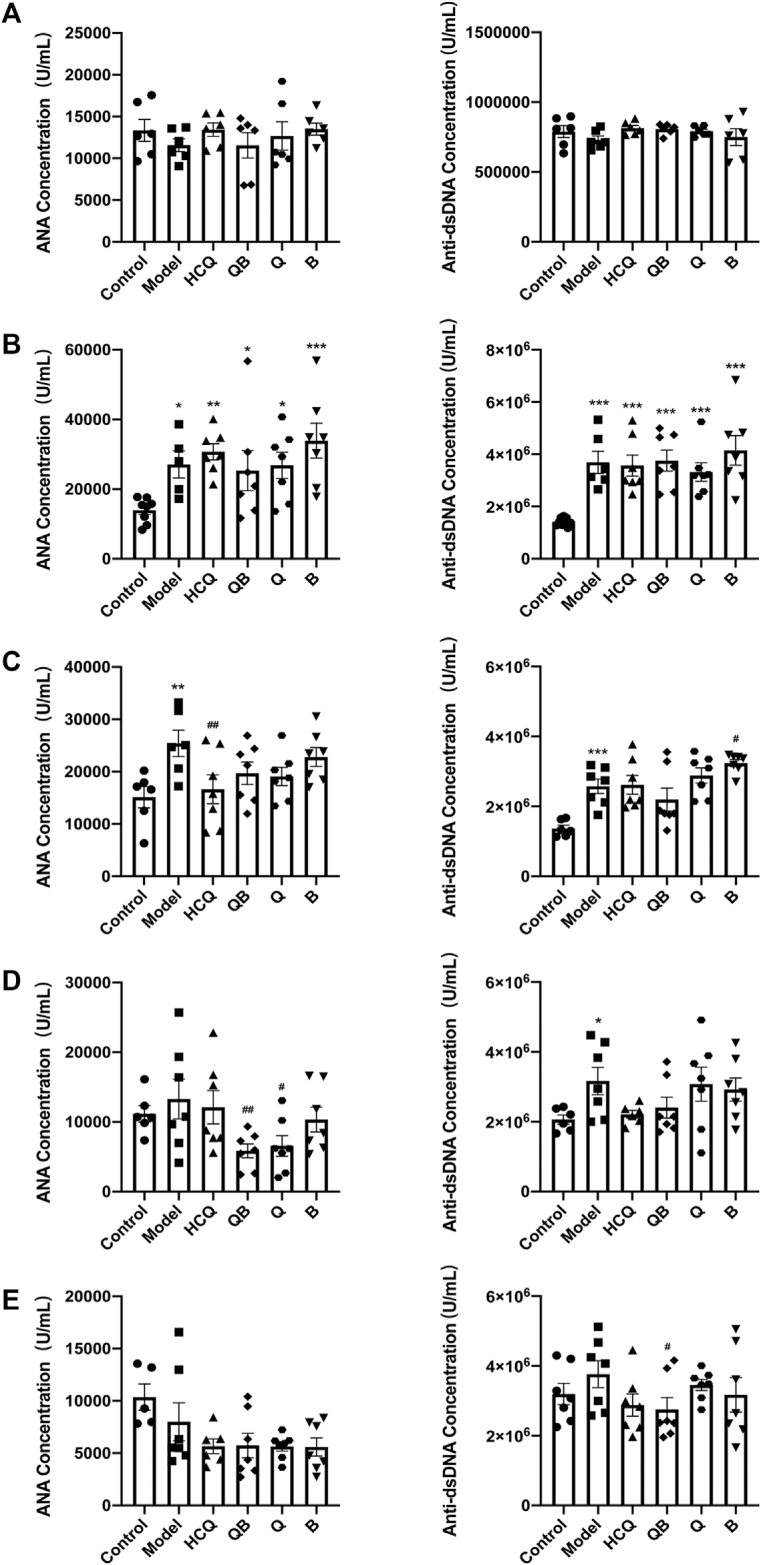
Qinghao-Biejia can reduce autoantibody production. **(A)** The antinuclear antibodies (ANA) and anti-double-stranded DNA antibodies (anti-dsDNA) level prior to modeling. **(B)** The ANA and anti-dsDNA level after modeling (compared with the control group, *p < 0.05, **p < 0.01, ***p < 0.001). **(C)** The ANA and anti-dsDNA level after 4 weeks of dosing. **(D)** The ANA and anti-dsDNA level after 8 weeks of dosing. **(E)** The ANA and anti-dsDNA level after 12 weeks of dosing. The data are expressed as the mean ± SEM. Model group compared with the control group, *p < 0.05, **p < 0.01, ***p < 0.001; the remaining groups compared with the model group, ^#^ *p* < 0.05, ^##^ *p* < 0.01.

The level of autoantibody production is a sensitive clinical indicator of SLE and is closely related to lupus activity (Alba et al., 2003), thus, the anti-dsDNA and ANA concentrations in serum were determined (Figure 2). Levels of anti-dsDNA and ANA in serum were detected at 4-week intervals. Prior to pristane injection, antibody levels in the serum of mice of each group remained consistent without significant difference. After 4 weeks of pristane injection, levels of anti-dsDNA and ANA in serum were detected to be significantly higher in mice from each SLE model group than those in the control group (Figures 2A,B). After 4 weeks of drug intervention, the ANA levels in the serum of mice in the HCQ group significantly reduced compared to those in the model group (Figure 2C). After 8 weeks of drug intervention, the levels of ANA were significantly lower in the QB and Q groups compared to the model group (Figure 2D). And after 12-week drug intervention, the levels of anti-dsDNA in the QB group significantly declined compared to the model group (Figure 2E). This indicated that QB can reduce autoantibodies production and thus inhibit lupus activity.

### Qinghao-Biejia can Improve Renal Injury

**FIGURE 3:**
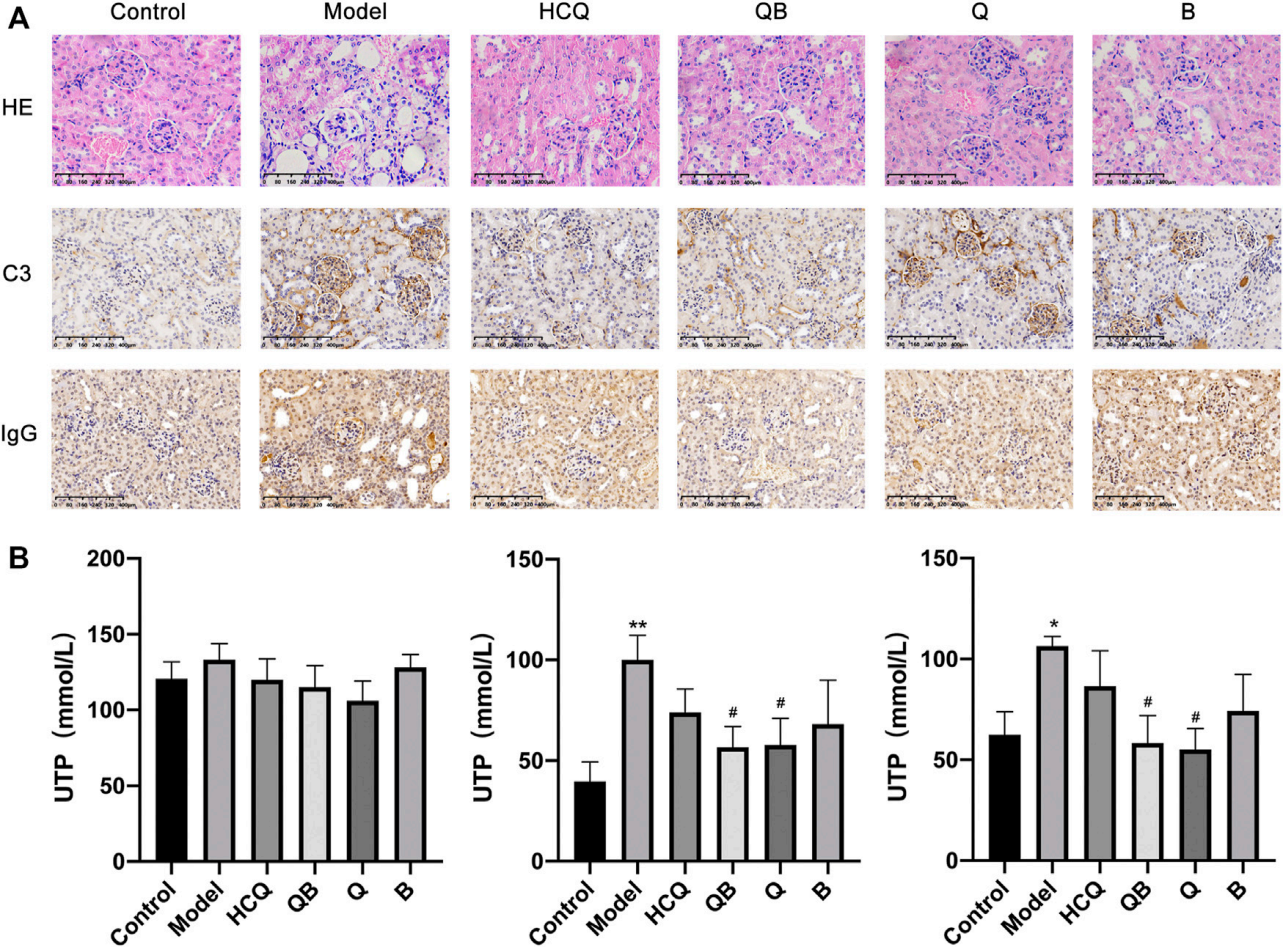
Qinghao-Biejia can improve renal injury. **(A)** Pathological changes in the spleen were assessed by HE staining and representative immunohistochemical staining for C3 and IgG (All images are x400). **(B)** The proteinuria levels of the mice (after 4 weeks of dosing, after 8 weeks of dosing, and after 12 weeks of dosing, respectively). The data are expressed as the mean ± SEM. Model group compared with the control group, *p < 0.05, **p < 0.01; the remaining groups compared with the model group, ^#^ *p* < 0.05.

HE staining was used to detect pathological changes in the kidney tissue at the end of the experiment. The results showed that SLE induced severe kidney injury in mice, including glomerular fibrosis, intra-globular cytopenia, swelling of tubular epithelial cells with glassy droplets, and inflammatory cell infiltration in the renal interstitium. Renal damage was alleviated to some extent after treatment with QB, Qinghao, Biejia, and HCQ. Immunohistochemical studies led to more significant morphological findings that C3 and IgG deposition in glomerular thylakoid capillary loops and interstitium in the model mice were more pronounced compared to control mice. SLE mice treated with QB and HCQ showed a noteworthy reduction in C3 and IgG deposition, whereas C3 and IgG deposition was slightly improved in ones treated with Qinghao and Biejia alone (Figure 3A).

Proteinuria is one of the major symptoms indicating the development of renal disease in SLE mice. At the beginning of the intervention (week 8, after 4 weeks of dosing), there was no significant difference in the proteinuria levels of the mice in each group. After 8 and 12 weeks of drug intervention, mice in the model group showed a significant increase in proteinuria compared to control group, while mice treated with QB and Qinghao showed a remarkable decrease in proteinuria levels (Figure 3B).

### Effect of Qinghao-Biejia on Aortic Atherosclerosis and Lipid Metabolism in Serum

**FIGURE 4:**
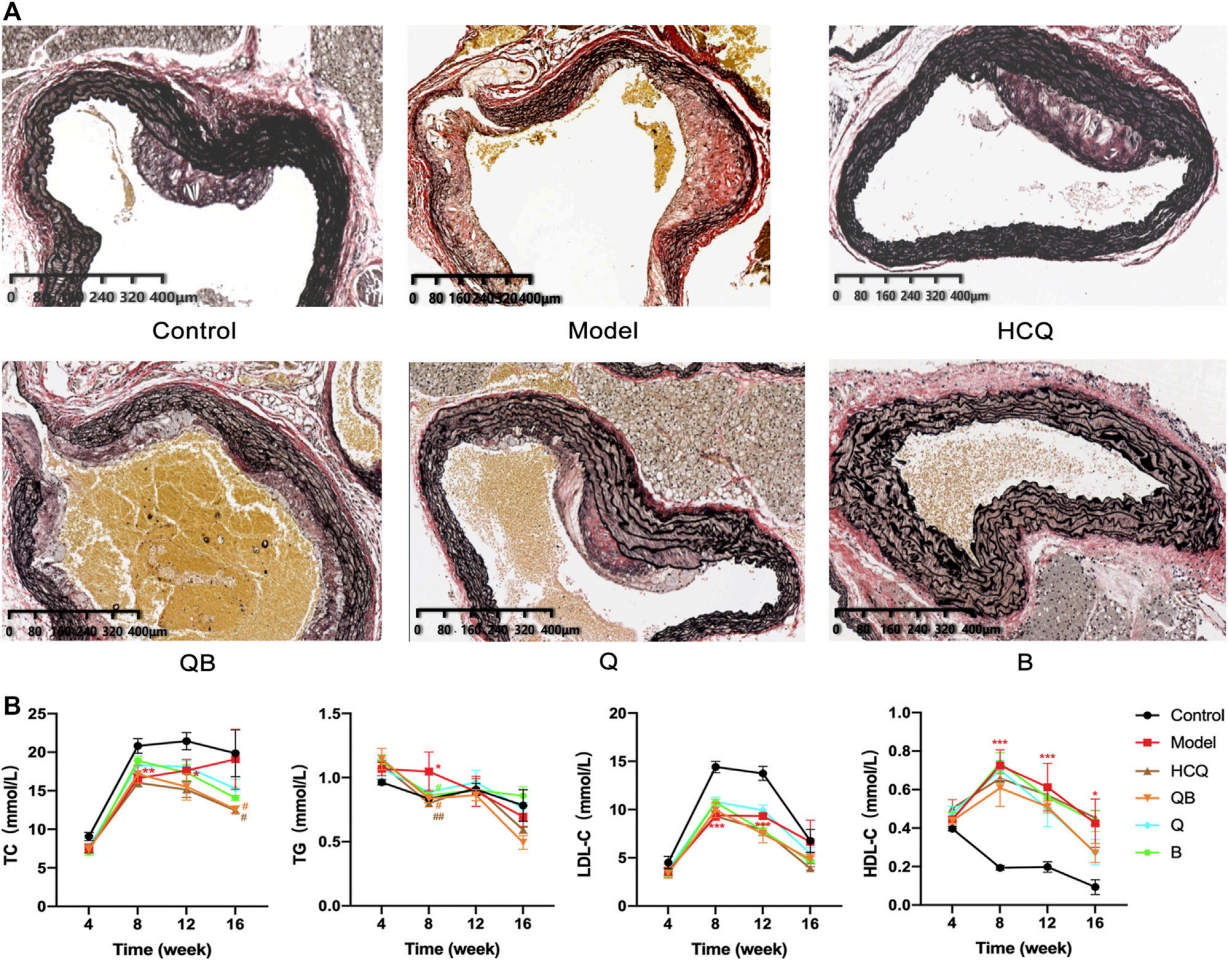
Effect of Qinghao-Biejia on aortic atherosclerosis and lipid metabolism in serum. **(A)** Representative images of EVG staining of aortic. **(B)** The level of total cholesterol (TC), triglyceride (TG), low density lipoprotein cholesterol (LDL-C) and high density lipoprotein cholesterol (HDL-C) in serum. The data are expressed as the mean ± SEM. Model group compared with the control group, *p < 0.05, **p < 0.01, ***p < 0.001; the remaining groups compared with the model group, ^#^ *p* < 0.05, ^##^ *p* < 0.01.

EVG staining is an effective method to visualize aortic atherosclerotic lesions. Observations showed that atheromatous plaque deposition was visible in the aorta of mice in the control group, with elastic fibers still intact. In contrast, aortic plaques in mice of the model group appeared to be more pronounced, with plaque bulging toward the intimal surface and compressing the tunica media, and tunica media thinning due to plaque compression, atrophy of smooth muscle cells, and destruction of elastic fibers. Arterial plaque deposition was conspicuously reduced in the HCQ, QB, Q, and B groups, and the elastic fibers of tunica media were more intact because of the low compression effect of arterial plaque (Figure 4A).

It is well known that high serum lipid levels are one of the main causes of the formation of atherosclerosis, so we measured TC, TG, HDL-C, and LDL-C in serum. In the analyses of lipid metabolism parameters, all six groups of mice were ApoE^−/−^ mice and all theoretically had higher lipid levels than normal C57BL/6 mice. After 16 weeks, the TC levels in the serum of mice of the QB and HCQ groups were significantly lower than those in the model group. At 8 weeks, the serum TG levels of mice in the QB, B, and HCQ groups were significantly lower than those in the model group, and at 16 weeks, there was a trend of decrease in the QB and HCQ groups, but without statistical difference. Throughout the course of administration, compared to the control group, HDL-C was significantly higher and LDL-C was lower in the remaining groups (Figure 4B).

### Effect of Qinghao-Biejia on Inflammatory Cytokines in Serum and Kidney

**FIGURE 5:**
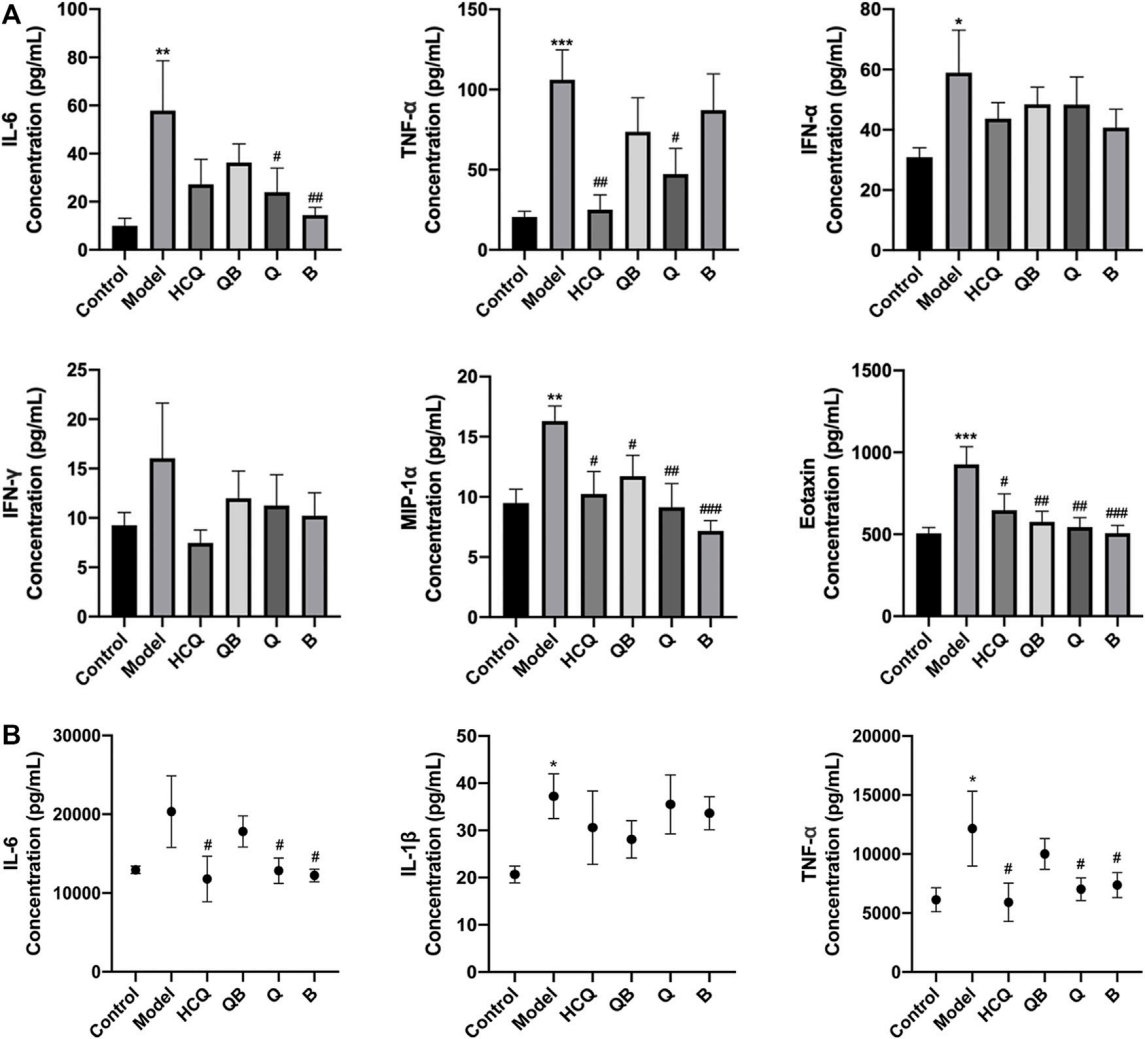
Effect of Qinghao-Biejia on inflammatory cytokines in serum and kidney. **(A)** The level of interleukin-6 (IL-6), tumour necrosis factor alpha (TNF-α), interferon alpha (IFN-α), interferon gamma (IFN-γ), macrophage inflammatory protein-1 alpha (MIP-1α), and eotaxin in serum. **(B)** The level of IL-6, interleukin-1 beta (IL-1β), TNF-α, and IFN-γ in kidney. The data are expressed as the mean ± SEM. Model group compared with the control group, *p < 0.05, **p < 0.01, ***p < 0.001; the remaining groups compared with the model group, ^#^ *p* < 0.05, ^##^ *p* < 0.01, ^###^ *p* < 0.001.

At 16 weeks, compared to the control group, the expression levels of IL-6, TNF-α, MIP-1α, IFN-α, and eotaxin in the serum were increased in model mice. HCQ was able to significantly reduce the expression levels of TNF-α, MIP-1α and eotaxin compared to the model group. QB remarkablly attenuated the increase on MIP-1α and eotaxin. Qinghao and Biejia effectively attenuated the increase of IL-6, MIP-1a, and eotaxin levels. In addition, drugs in each group partially alleviated this trend of IFN-α and IFN-γ without significant difference (Figure 5A).

Considering that inflammatory responses play a crucial role in the progression of lupus-like disease, we measured the expression levels of IL-6, IL-1β, and TNF-α in kidney tissues. At 16 weeks, the expression levels of TNF-α and IL-1β in the kidneys were distinctly increased in model group compared to the control. Compared to the model group, the effects of Qinghao, Biejia, and HCQ obviously inhibited the elevated expression levels of IL-6 and TNF-α. There was a trend of partial reduction in the expression levels of IL-1β after drug administration, but there was no statistical difference (Figure 5B).

### Qinghao-Biejia can Partly Inhibit HMGB1/TLR4 Pathway in Kidneys

**FIGURE 6:**
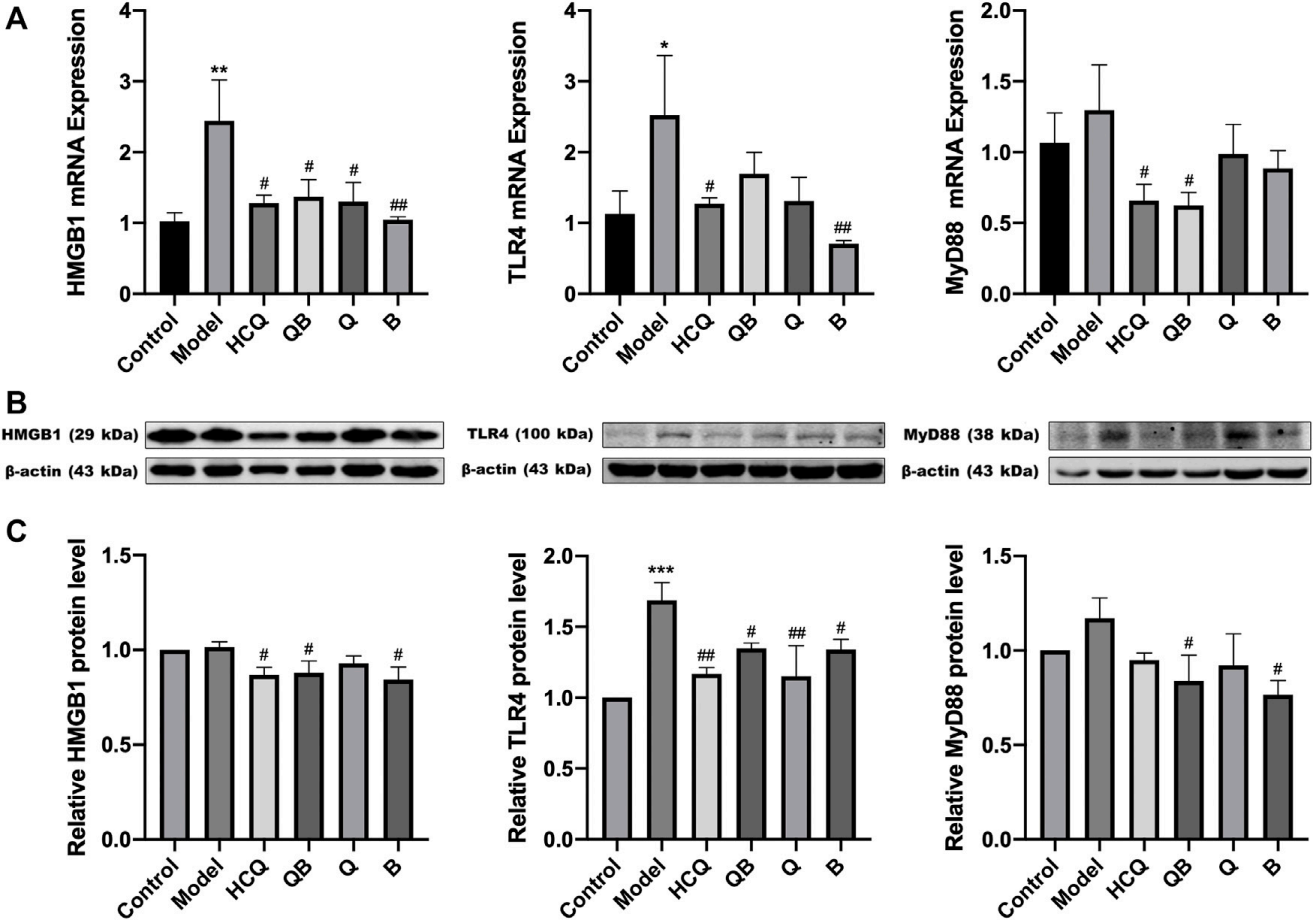
Qinghao-Biejia can partly inhibit HMGB1/TLR4 pathway in kidneys. **(A)** The mRNA expression level of high mobility group box 1 protein (HMGB1), toll like receptor 4 (TLR4), and myeloid differentiation primary response 88 (MyD88) determined by RT-qPCR. **(B)** Band diagrams of HMGB1, TLR4, and MyD88 in kidney tissue of mice determined by western blot. **(C)** The expression level of HMGB1, TLR4, and MyD88 in kidney tissue of mice determined by western blot. Model group compared with the control group, *p < 0.05, **p < 0.01, ***p < 0.001; the remaining groups compared with the model group, ^#^ *p* < 0.05, ^##^ *p* < 0.01.

To explore the molecular mechanisms of QB-mediated nephroprotection in SLE mice, RT-qPCR, and Western Blot assays were required at the end of the experiment to confirm the expression of HMGB1, TLR4, and MyD88. The expression levels of HMGB1 and TLR4 mRNA in the kidneys of the model group remarkably increased, whereas each dosing group including QB, Q, B, and HCQ was able to significantly decrease the expression level of mRNA in HMGB1/TLR4 signaling (Figure 6A).

Interestingly, combining the above proteinuria assay and renal pathological results, we found that the kidney damage was more severe in the model group. At the same time, the protein expression of HMGB1, TLR4, and MyD88 were higher in the model group. However, the mice had less renal damage with the treatment of QB, and the corresponding protein expression of HMGB1/TLR4 tended to decrease (Figures 6B,C).

## Discussion

SLE is a complex and heterogeneous systemic autoimmune disease that affects the life and health of a large population. Recent reports indicated that the overall global incidence of SLE ranges between 1.5 and 11 per 100,000 individuals-years and the global prevalence ranges from 13 to 7713.5 per 100,000 individuals (Barber et al., 2021). Multiple meta-analyses indicated that patients with SLE exhibited dramatical increased subclinical atherosclerosis compared to controls (Wu et al., 2016; Henrot et al., 2018). Therefore, new drugs for SLE and atherosclerosis complications still need to be identified. In this research, we applied ApoE^−/−^ mice to establish a model of SLE combined with atherosclerosis by intraperitoneal injection of pristane, and evaluated the effects of QB, Qinghao, Biejia, and a representative western drug, HCQ, on SLE combined with atherosclerosis.

Pristane triggers autoantibody production that most closely resembles the autoantibody spectrum observed in human SLE, and pristine is able to induce a wide variety of autoantibodies specific to or associated with SLE in mice (Freitas et al., 2017). Mice administered pristane intraperitoneally develop monoclonal antibody-rich ascitic fluid, local chronic inflammation and rheumatoid erosive arthritis, as well as autoantibodies and clinical manifestations similar to those of SLE (Satoh et al., 1995; Freitas et al., 2017). ApoE is a component of VLDL and HDL and is involved in cholesterol transport. ApoE^−/−^ mice show impaired plasma lipoproteins clearance and they develop atherosclerosis in a short time (Kolovou et al., 2008). The present study used a model of ApoE^−/−^ C57BL/6 mice in which pristane administration resulted in SLE combined with atherosclerosis, presenting reduced spontaneous activity, marked alopecia, splenomegaly, and significantly higher levels of ANA and anti-dsDNA, in agreement with previous reports (Chen et al., 2016b). In addition, pathological results showed evident atheromatous plaques in the aorta of the mice with or without pristane injection. This suggests that the mice models of SLE combined with atherosclerosis in this study was successfully established, which lays the foundation for further study of the mechanism.

Jieduquyuziyin prescription has been used effectively and exclusively for almost 20 years in the treatment of SLE at the Chinese Traditional Medicine Hospital of Zhejiang Province, China (Ji et al., 2020). The whole prescription consists of 10 traditional Chinese herbs including Qinghao, Biejia, Chishao, Xuejicao, etc. Many animals experimental results and clinical data prove that it can modulate immunity, control inflammation, reduce the incidence of infections, as well as lessen the side effects of western drugs (Wen et al., 2007; Shui et al., 2015). Qinghao and Biejia are the core components of this prescription. Artemisinin, the main component of Qinghao, can inhibit the production of serum anti-ds-DNA in lupus-like mice and has an inhibitory effect on the secretion of TNF-α in serum (Wu et al., 2010). Trionyx sinensis polysaccharides, an extract of Biejia, can significantly improve non-specific immune function and cellular immune function. Due to the interdependent, interactional and mutual relationships among multiple herbs, it is difficult to identify the real effects of single herb in prescriptions and draw accurate conclusions from the study of prescription, therefore, the study of core herb pairs can precisely fill these gaps.

SLE has multiple clinical manifestations. Flares, unpredictable course and clinical heterogeneity are characteristics of SLE. Tissue damage caused by autoantibodies and immune complex depositions occurs in kidneys, vessels, heart and other organs, with kidney damage being the most severe, leading to significant mortality (Fanouriakis et al., 2021). LN remains a major therapeutic challenge (Parikh et al., 2020). In the present study, pristine-induced lupus mice treated with QB showed obvious improvements in renal architecture, as reflected by glomerular size, the extent of thylakoid matrix expansion and glomerular epithelial remnants. Meanwhile, QB dramatically alleviated the swelling of the spleen and reduced the splenic index in mice, which is one of the most important indicators of SLE progression. Proteinuria is the most representative indicator of renal inflammation, and the key pathological feature is the damage of the glomerular filtration barrier (GFB), which reflects the progression of renal disease (Okada et al., 2017). In the present study, QB significantly improved the symptoms of pristine-induced lupus mice and inhibited the progression of proteinuria after 12 weeks of dosing. Numerous studies have suggested that autoantibody production and glomerular immune deposit formation are vital initial events in the pathogenesis of lupus (Waldman and Madaio, 2005). The levels of ANA and anti-dsDNA in serum are specific for SLE and are widely used to help establish the diagnosis of SLE and predict lupus activity (Pisetsky and Lipsky, 2020). The immune complex deposition can trigger a series of events that lead to kidney inflammation and injury (Nie et al., 2016). In pristine-induced lupus mice, we suggested that QB treatment decreased the levels of serum ANA and anti-dsDNA. It also dramatically reduced the deposition of immune complex complement C3 and IgG in the kidney. All of these indicated that QB had an ameliorative effect on the disease.

More notably, organ damage in SLE results from the infiltration of activated T cells into susceptible organs and the deposition of immune complexes. Defective immune regulation and increased maturation of antigen-presenting cells, as well as uncontrolled lymphocyte activation are influenced by cytokines (Apostolidis et al., 2011). Thus, cytokines are intimately involved in every step of the pathogenesis of SLE. Cell death is caused by the attack of complement terminal membrane and cytotoxic T cells on complexes, while inflammation is mainly induced by monocytes/macrophages. In combination with immune complexes, monocytes/macrophages produce a number of pro-inflammatory cytokines, such as IL-1, IL-6, TNF, etc. It has been reported that IL-1, IL-6, IL-18, and TNF are highly overexpressed in the kidneys of mice and humans with LN (Aringer and Smolen, 2005), and thus directly linked to inflammation. In addition to these inflammatory pathways, the detection of immune complexes leads to the production of chemokines and type I interferons which play an immunomodulatory role (Aringer, 2020). The results of the present study showed that QB could reduce the expression of pro-inflammatory factors such as IL-6, IL-1β and TNF-α, as well as cytokines such as IFN-α, IFN-γ, MIP-1α, and eotaxin to some extent.

SLE carries a significant risk of CVD. The pathogenesis of CVD in SLE is not fully understood, but the inflammatory nature of SLE is thought to be a critical factor in accelerating atherosclerosis. Atherosclerosis is characterized as an immunoinflammatory disorder. Dysregulated immune repertoire in SLE causes inflammation that induces endothelial dysfunction and vascular injury and leads to monocyte chemotaxis to the subintima. In the subintima, monocytes transform into macrophages and uptake of oxidatively modified lipids, forming foam cells that eventually manifest as fatty streak lesions (Reiss, 2009). Systemic inflammation may lead to dyslipidemia in the body with dysfunction of TG, TC, LDL-C, and HDL-C (Reiss et al., 2021). In this study, the results showed that the control and model groups had obvious deposits of atherosclerotic plaque, which were dramatically reduced after QB treatment, with more intact intima-media elastin fibers. This could be related to the results that abnormal lipid profile get moderated and serum inflammatory factors such as IL-6, TNF-a decreased.

HMGB1 is a non-histone nuclear protein that acts as an alarm agent to drive the pathogenesis of inflammatory and autoimmune diseases. Currently, it has been demonstrated that extracellular HMGB1 levels are dramatically elevated in SLE patients and are strongly correlated with lupus activity (Yang et al., 2020). It has been confirmed that HMGB1 contributes to mesangial cell proliferation and extracellular matrix deposition (Feng et al., 2020). Notably, extracellular HMGB1 is not only predominantly located in glomerular mesangial cells, but also has an abnormally increased expression in glomerular endothelial cells in LN. In general, extracellular HMGB1 mediated cellular injury and induced inflammatory cytokine production by binding to the cell surface receptor TLR4 and triggering related intracellular pathways (Janko et al., 2014). A recent study demonstrated that extracellular HMGB1 induced endothelial glycocalyx shedding and glomerular endothelial cells damage in LN by activating the downstream signaling pathway TLR4/MyD88 (Yu et al., 2021). Not only that, but in SLE, HMGB1 can be a component of the immune complex containing anti-DNA, as it interacts with DNA (Harris et al., 2012). The present experiment corroborates these claims, and it also implies that QB may be able to inhibit the HMGB1/TLR4/MyD88 pathway, thereby reduce autoantibody production, inhibit the release of inflammatory factors, and improve kidney damage.

Our previous study reported that QB could inhibit the production of IL-6 and secretion of IFN-γ in the serum of MRL/lpr mice, and significantly improve the pathologic lesions of LN in mice (Gan et al., 2015). It is on the basis of these results and the high incidence of atherosclerosis in SLE patients that the present study was conducted to investigate the effect of QB on SLE combined with atherosclerosis by establishing a mouse model of SLE combined with atherosclerosis. In this study, not only the levels of inflammatory factors in the serum and kidney were measured, but also the deposition of immune complexes such as C3 and IgG in the kidney were observed by immunohistochemistry, and the lesions of the aorta were also focused on. It is worth mentioning that the present study also made a preliminary exploration of the drug targets. It is hoped to provide an experimental basis for the clinical application of QB.

## Conclusion

In conclusion, the present study demonstrated for the first time that QB can reduce the levels of autoantibody, improve kidney damage, as well as regulate lipid metabolism and improve aortic damage. More interestingly, QB-treated mice showed significant improvements not only in inflammatory factor secretion, but also in HMGB1/TLR4/MyD88 pathways. These new findings support the hypothesis that QB plays a role in reducing the inflammatory response and disease progression in SLE combined with atherosclerosis. Although the exact mechanism has not been fully elucidated, the HMGB1/TLR4/MyD88 signaling pathway appears to be a new target for SLE combined with atherosclerosis treatment.

## Data Availability

The original contributions presented in the study are included in the article/Supplementary Material, further inquiries can be directed to the corresponding authors.

## Supplementary Material

Supplementary Figure S1 Experimental protocol.

Supplementary Figure S2 Total ion current chromatograms of Qinghao-Biejia decoction by UPLC-TOF-MS. **(A)** negative ion mode. **(B)** positive ion mode.

Supplementary Table S1 Chemical composition analy sis of Qinghao-Biejia decoction by UPLC-TOF-MS under negative and positive ion mode. **(A)** negative ion mode. **(B)** positive ion mode.

## Glossary

SLE: systemic lupus erythematosus
CVD: cardiovascular disease
TCM: traditional Chinese medicine
LN: lupus nephritis
QB: Qinghao-Biejia herb pair
HMGB1: high mobility group box 1 protein
TLR4: toll like receptor 4
MyD88: myeloid differentiation primary response 88
NF-κB: nuclear factor kappa-B
HCQ: hydroxychloroquine
UPLC-TOF-MS: ultrahigh-performance liquid chromatography time of flight mass spectrometry
ESI: electrospray ionization
m/z: mass-to-charge ratio
SPF: specific pathogen free
Q group: Qinghao group
B group: Biejia group
Anti-dsDNA: anti-double-stranded DNA antibodies
ANA: antinuclear antibodies
ELISA: enzyme-linked immunosorbent assay
TC: total cholesterol
TG: triglyceride
LDL-C: low density lipoprotein cholesterol
HDL-C: high density lipoprotein cholesterol
IHC: immunohistochemistry
IL-6: interleukin-6
TNF-α: tumour necrosis factor alpha
MIP-1α: macrophage inflammatory protein-1 alpha
IFN-α: interferon alpha
IFN-γ: interferon gamma
IL-1β: interleukin-1 beta
RT-qPCR: real-time quantitative polymerase chain reaction
Ct: cycle number at which transcripts could be detected
RIPA: radio immunoprecipitation assay
ANOVA: analysis of variance
GFB: glomerular filtration barrier
GAPDH: glyceraldehyde-3-phosphate dehydrogenase
