# Supplementary figures and images for: Qinghao-Biejia Herb Pair Alleviates Pristane-Induced Lupus-Like Disease and Associated Renal and Aortic Lesions in ApoE−/− Mice
Source: Front Pharmacol. 2022 Apr 29;13:897669. doi: 10.3389/fphar.2022.897669 (PMC9100684; doi:10.3389/fphar.2022.897669)

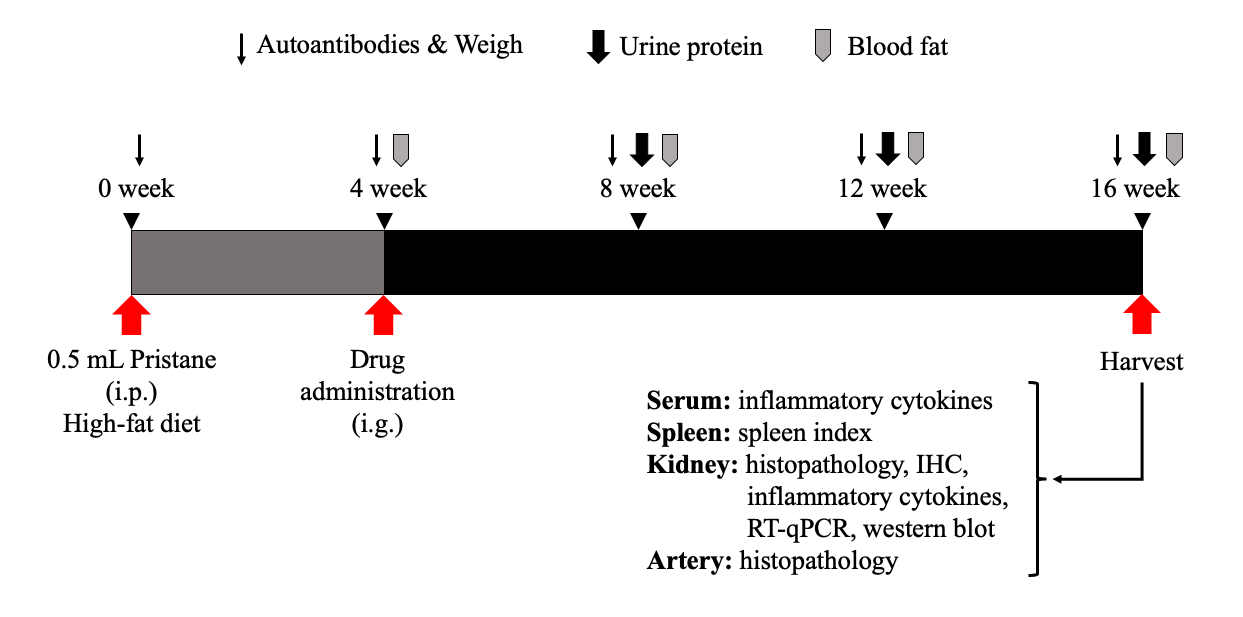

Supplement: Supplementary file 1 [file Image1.TIFF]

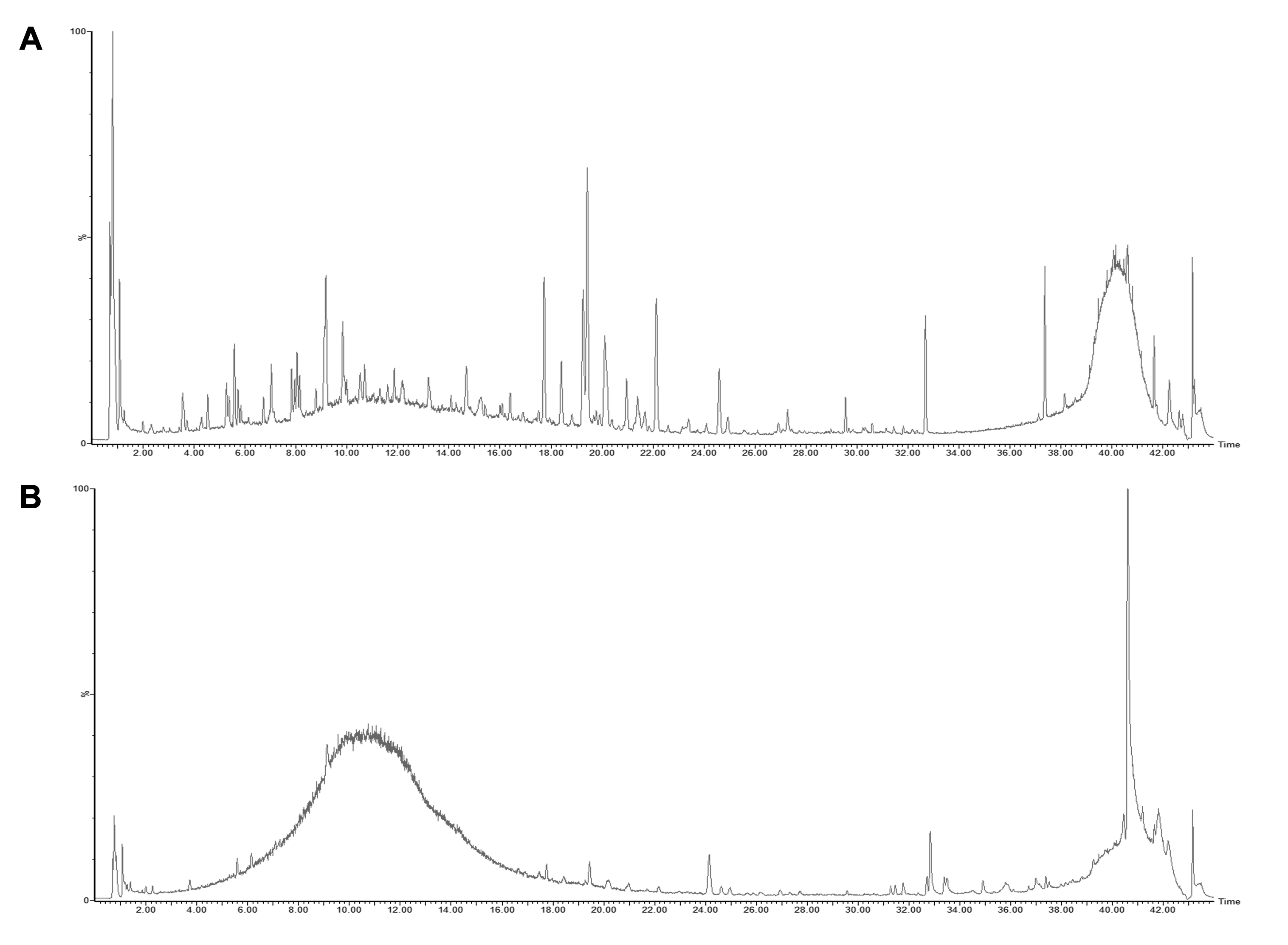

Supplement: Supplementary file 3 [file Image2.TIF]
