# Supplementary material for: Qinghao-Biejia Herb Pair Alleviates Pristane-Induced Lupus-Like Disease and Associated Renal and Aortic Lesions in ApoE−/− Mice
Source: Front Pharmacol. 2022 Apr 29;13:897669. doi: 10.3389/fphar.2022.897669 (PMC9100684; doi:10.3389/fphar.2022.897669)
Supplement: Supplementary file 2 [file Table1.DOCX]

| \| **Table S1-A Chemical composition analysis of Qinghao-Biejia decoction by UPLC-TOF/MS under negative ion mode.** \| \| \| \| \| \| \| \| \| --- \| --- \| --- \| --- \| --- \| --- \| --- \| --- \| \| Compound \| Retention time/min \| Name \| Chemical Formula \| Molecular Mass/Da \| m/z Observation \| δ/ppm \| Adduct \| \| 1 \| 0.73 \| Arginine \| C_6_H_14_N_4_O_2_ \| 174.112 \| 173.106 \| 9.2 \| -H \| \| 2 \| 0.79 \| Pentose \| C_5_H_10_O_5_ \| 150.053 \| 195.052 \| 6.9 \| +HCOO \| \| 3 \| 0.81 \| Galactose \| C_6_H_12_O_6_ \| 180.063 \| 179.058 \| 9.3 \| -H \| \| 4 \| 0.84 \| Quercetagetin \| C_15_H_10_O_8_ \| 318.038 \| 363.033 \| -7.6 \| +HCOO \| \| 5 \| 1.08 \| 3,4-Dihydroxy-6,7,3',4'-tetramethoxyflavone \| C_19_H_20_O_7_ \| 360.121 \| 405.122 \| 6.8 \| +HCOO \| \| 6 \| 4.88 \| Aurantiamide acetate \| C_27_H_28_N_2_O_4_ \| 444.205 \| 443.195 \| -6.8 \| -H \| \| 7 \| 5.58 \| Scopolin \| C_16_H_18_O_9_ \| 354.095 \| 399.097 \| 8.1 \| +HCOO, +Cl \| \| 8 \| 9.11 \| Scopoletin \| C_10_H_8_O_4_ \| 192.042 \| 191.037 \| 8.6 \| -H \| \| 9 \| 9.52 \| Artemitin \| C_20_H_20_O_8_ \| 388.116 \| 387.110 \| 2.4 \| -H \| \| 10 \| 9.85 \| 5,6-Dimethoxy-7-hydroxycoumarin \| C_11_H_10_O_5_ \| 222.053 \| 221.047 \| 8.5 \| -H \| \| 11 \| 10.25 \| Salicylic acid \| C_7_H_6_O_3_ \| 138.032 \| 137.026 \| 8.1 \| -H \| \| 12 \| 10.56 \| Rutin \| C_27_H_30_O_16_ \| 610.153 \| 609.149 \| 4.7 \| -H \| \| 13 \| 10.59 \| Isoquercitrin \| C_21_H_20_O_12_ \| 464.095 \| 463.093 \| 10 \| -H \| \| 14 \| 11.28 \| Patuletin-3-O-glucoside \| C_22_H_22_O_13_ \| 494.106 \| 493.102 \| 7.1 \| -H \| \| 15 \| 12.34 \| Kaempferol 3-O-β-D-glucoside \| C_21_H_20_O_11_ \| 448.101 \| 447.097 \| 7.8 \| -H \| \| 16 \| 14.90 \| Benzyl D-2-methylbutyrate \| C_12_H_16_O_2_ \| 192.115 \| 237.115 \| 5.8 \| +HCOO \| \| 17 \| 15.25 \| Phenyl acetate \| C_8_H_8_O_2_ \| 136.052 \| 181.052 \| 8.1 \| +HCOO \| \| 18 \| 15.38 \| Artemisinin \| C_15_H_22_O_5_ \| 282.147 \| 327.145 \| 1.3 \| +HCOO \| \| 19 \| 15.95 \| β-Santonin \| C_15_H_18_O_3_ \| 246.126 \| 291.126 \| 9 \| +HCOO \| \| 20 \| 17.22 \| 1,8-Cineole \| C_10_H_18_O \| 154.136 \| 199.136 \| 9.5 \| +HCOO \| \| 21 \| 17.87 \| Artemisitene \| C_15_H_20_O_5_ \| 280.131 \| 279.124 \| 0.2 \| -H \| \| 22 \| 18.41 \| Qinghaosu-Ⅳ \| C_15_H_22_O_5_ \| 282.147 \| 281.142 \| 9.9 \| -H \| \| 23 \| 18.87 \| Fenchone \| C_10_H_16_O \| 152.120 \| 197.120 \| 9.9 \| +HCOO \| \| 24 \| 19.42 \| Arteamisinine III \| C_15_H_22_O_4_ \| 266.152 \| 265.147 \| 7.3 \| -H \| \| 25 \| 20.58 \| Arteamisinine I \| C_13_H_18_O_2_ \| 206.131 \| 251.129 \| 2 \| +HCOO \| \| 26 \| 21.30 \| Isobornyl acetate \| C_12_H_20_O_2_ \| 196.146 \| 241.145 \| 2.5 \| +HCOO \| \| 27 \| 23.28 \| ψ-santonin \| C_15_H_20_O_4_ \| 264.136 \| 263.131 \| 8.8 \| -H \| \| 28 \| 24.59 \| Artemisinin B \| C_15_H_20_O_3_ \| 248.141 \| 247.136 \| 8 \| -H \| \| 29 \| 24.94 \| Casticin \| C_19_H_18_O_8_ \| 374.100 \| 373.095 \| 5.8 \| -H \| \| 30 \| 25.24 \| Artemisilactone \| C_15_H_22_O_3_ \| 250.157 \| 249.152 \| 9 \| -H \| \| 31 \| 28.66 \| Methyl arteannuate \| C_16_H_24_O_2_ \| 248.178 \| 293.178 \| 7.2 \| +HCOO \| \| 32 \| 28.77 \| Farnesyl Acetate \| C_17_H_28_O_2_ \| 264.209 \| 309.206 \| -2.4 \| +HCOO \| \| 33 \| 32.65 \| Artemisinic Alcohol \| C_15_H_28_O \| 224.214 \| 269.213 \| 4 \| +HCOO \| \| 34 \| 32.69 \| (+)-artemisinic acid \| C_15_H_22_O_2_ \| 234.162 \| 233.157 \| 9.5 \| -H \| \| 35 \| 34.61 \| 5,3’,4’-Trihydroxy-6,7-dimethoxyflavone \| C_17_H_14_O_7_ \| 330.074 \| 329.065 \| -5.8 \| -H \| \| 36 \| 42.36 \| Ethyl Palmitate \| C_18_H_36_O_2_ \| 284.272 \| 283.267 \| 8.3 \| -H \| \| 37 \| 42.56 \| Ethyl laurate \| C_14_H_28_O_2_ \| 228.209 \| 227.204 \| 9.8 \| -H \| \| 38 \| 43.13 \| Cysteine \| C_3_H_7_NO_2_S \| 121.020 \| 166.019 \| 7.2 \| +HCOO \|   **Table S1-B Chemical composition analysis of Qinghao-Biejia decoction by UPLC-TOF/MS under positive ion mode.** | | | | | | | |
| --- | --- | --- | --- | --- | --- | --- | --- | --- | --- | --- | --- | --- | --- | --- | --- | --- | --- | --- | --- | --- | --- | --- | --- | --- | --- | --- | --- | --- | --- | --- | --- | --- | --- | --- | --- | --- | --- | --- | --- | --- | --- | --- | --- | --- | --- | --- | --- | --- | --- | --- | --- | --- | --- | --- | --- | --- | --- | --- | --- | --- | --- | --- | --- | --- | --- | --- | --- | --- | --- | --- | --- | --- | --- | --- | --- | --- | --- | --- | --- | --- | --- | --- | --- | --- | --- | --- | --- | --- | --- | --- | --- | --- | --- | --- | --- | --- | --- | --- | --- | --- | --- | --- | --- | --- | --- | --- | --- | --- | --- | --- | --- | --- | --- | --- | --- | --- | --- | --- | --- | --- | --- | --- | --- | --- | --- | --- | --- | --- | --- | --- | --- | --- | --- | --- | --- | --- | --- | --- | --- | --- | --- | --- | --- | --- | --- | --- | --- | --- | --- | --- | --- | --- | --- | --- | --- | --- | --- | --- | --- | --- | --- | --- | --- | --- | --- | --- | --- | --- | --- | --- | --- | --- | --- | --- | --- | --- | --- | --- | --- | --- | --- | --- | --- | --- | --- | --- | --- | --- | --- | --- | --- | --- | --- | --- | --- | --- | --- | --- | --- | --- | --- | --- | --- | --- | --- | --- | --- | --- | --- | --- | --- | --- | --- | --- | --- | --- | --- | --- | --- | --- | --- | --- | --- | --- | --- | --- | --- | --- | --- | --- | --- | --- | --- | --- | --- | --- | --- | --- | --- | --- | --- | --- | --- | --- | --- | --- | --- | --- | --- | --- | --- | --- | --- | --- | --- | --- | --- | --- | --- | --- | --- | --- | --- | --- | --- | --- | --- | --- | --- | --- | --- | --- | --- | --- | --- | --- | --- | --- | --- | --- | --- | --- | --- | --- | --- | --- | --- | --- | --- | --- | --- | --- | --- | --- | --- | --- | --- | --- | --- | --- | --- | --- | --- | --- | --- | --- | --- | --- | --- | --- | --- | --- | --- | --- | --- | --- | --- | --- | --- | --- | --- | --- | --- | --- | --- | --- | --- |
| Compound | Retention time/min | Name | Chemical Formula | Molecular Mass/Da | m/z Observation | δ/ppm | Adduct |
| 1 | 0.79 | Galactose-1 | C_6_H_12_O_6_ | 180.063 | 203.053 | 2.4 | +Na |
| 2 | 0.80 | Valine | C_5_H_11_NO_2_ | 117.079 | 118.086 | -2.4 | +H, +K |
| 3 | 0.82 | Scopolin | C_16_H_18_O_9_ | 354.095 | 355.103 | 1.6 | +H |
| 4 | 0.82 | Proline | C_5_H_9_NO_2_ | 115.063 | 116.071 | 2.2 | +H |
| 5 | 0.84 | Glutamic acid | C_5_H_9_NO_4_ | 147.053 | 148.061 | 4.8 | +H |
| 6 | 2.00 | Hydrocinnamic acid | C_9_H_11_NO_2_ | 165.079 | 166.087 | 1.8 | +H |
| 7 | 5.28 | Coumarin | C_9_H_6_O_2_ | 146.037 | 147.044 | 1.1 | +H |
| 8 | 6.73 | Isoleucine | C_6_H_13_NO_2_ | 131.095 | 132.102 | 1.5 | +H |
| 9 | 7.21 | Citronellol | C_10_H_20_O | 156.151 | 195.114 | -4.5 | +K |
| 10 | 9.12 | Scopoletin | C_10_H_8_O_4_ | 192.042 | 193.050 | 0.1 | +H |
| 11 | 9.12 | Phenyl acetate | C_8_H_8_O_2_ | 136.052 | 137.060 | 2.3 | +H |
| 12 | 9.84 | Phenylacetic acid | C_8_H_8_O_2_ | 136.052 | 137.060 | 2.7 | +H |
| 13 | 9.86 | 5,6-Dimethoxy-7-hydroxycoumarin | C_11_H_10_O_5_ | 222.053 | 223.061 | 2.9 | +H |
| 14 | 10.58 | Rutin | C_27_H_30_O_16_ | 610.153 | 611.162 | 2.5 | +H |
| 15 | 11.06 | Kaempferol 3-O-β-D-glucoside | C_21_H_20_O_11_ | 448.101 | 449.110 | 4.6 | +H |
| 16 | 11.06 | Kaempferol | C_15_H_10_O_6_ | 286.048 | 287.056 | 2.7 | +H |
| 17 | 11.29 | Patuletin-3-O-glucoside | C_22_H_22_O_13_ | 494.106 | 495.115 | 4.2 | +H |
| 18 | 11.30 | Patuletin | C_16_H_12_O_8_ | 332.053 | 333.061 | 1.2 | +H |
| 19 | 11.80 | 6-Hydroxykaempferol 3-O-β-D-glucoside | C_22_H_22_O_12_ | 478.111 | 479.119 | 2.1 | +H |
| 20 | 11.97 | 5,7,8,3'-tetrahydroxy-3,4'-dimethoxyflavone | C_17_H_14_O_8_ | 346.069 | 347.077 | 3.5 | +H |
| 21 | 12.74 | 3-O-Methylquercetin | C_16_H_12_O_7_ | 316.058 | 317.067 | 3.9 | +H |
| 22 | 13.26 | Scoparone | C_11_H_10_O_4_ | 206.058 | 207.066 | 4.1 | +H |
| 23 | 14.32 | β-Santonin | C_15_H_18_O_3_ | 246.126 | 247.133 | 1.1 | +H |
| 24 | 14.32 | ψ-Santonin | C_15_H_20_O_4_ | 264.136 | 265.144 | 0.3 | +H, +K, +Na |
| 25 | 16.75 | Isobornyl acetate | C_12_H_20_O_2_ | 196.146 | 219.136 | 2.6 | +Na |
| 26 | 17.09 | (±)-2-methylene-6,6-dimethyl-bicyclo[3.1.1]heptane | C_10_H_16_ | 136.125 | 137.133 | 3.9 | +H |
| 27 | 17.75 | Artemisinin | C_15_H_22_O_5_ | 282.147 | 305.136 | -0.6 | +Na |
| 28 | 18.43 | Qinghaosu-Ⅳ | C_15_H_22_O_5_ | 282.147 | 305.136 | -1.5 | +Na |
| 29 | 18.47 | Epoxyarteannuinic acid | C_15_H_22_O_3_ | 250.157 | 251.163 | -2.9 | +H |
| 30 | 20.99 | Quercetin | C_15_H_10_O_7_ | 302.043 | 303.051 | 3.3 | +H |
| 31 | 20.99 | Eupatin | C_18_H_16_O_8_ | 360.085 | 361.092 | 0.4 | +H, +Na |
| 32 | 22.06 | Benzyl D-2-methylbutyrate | C_12_H_16_O_2_ | 192.115 | 193.123 | 1.8 | +H |
| 33 | 22.98 | Artemisilactone | C_15_H_22_O_3_ | 250.157 | 273.147 | 4.6 | +Na, +K |
| 34 | 24.15 | Cumic alcohol | C_10_H_14_O | 150.104 | 151.112 | 2.1 | +H |
| 35 | 24.15 | Artemisinin B | C_15_H_20_O_3_ | 248.141 | 249.149 | 1.1 | +H, +K, +Na |
| 36 | 24.30 | 5,4'-dihydroxy-3,6,7-trimethoxyflavone | C_18_H_16_O_7_ | 344.090 | 345.098 | 1.8 | +H |
| 37 | 24.96 | Isorhamnetin | C_16_H_12_O_7_ | 316.058 | 317.066 | 0 | +H |
| 38 | 24.96 | Casticin | C_19_H_18_O_8_ | 374.100 | 375.107 | -0.6 | +H, +Na |
| 39 | 25.88 | Isopropyl salicylate | C_10_H_12_O_3_ | 180.079 | 181.087 | 3.9 | +H |
| 40 | 26.94 | 6,7-Dehydroartemisinic acid | C_15_H_20_O_2_ | 232.146 | 233.154 | 0.1 | +H |
| 41 | 26.98 | 2-Benzyloctanal | C_15_H_22_O | 218.167 | 219.175 | 1.3 | +H |
| 42 | 27.71 | Arteamisinine III | C_15_H_22_O_4_ | 266.152 | 267.160 | 1.7 | +H, +Na |
| 43 | 27.71 | Arteamisinine I | C_13_H_18_O_2_ | 206.131 | 207.138 | 0.5 | +H |
| 44 | 28.54 | Artemitin | C_20_H_20_O_8_ | 388.116 | 389.124 | 1.6 | +H |
| 45 | 28.93 | Bornyl isovalerate | C_15_H_26_O_2_ | 238.193 | 261.181 | -4.6 | +Na |
| 46 | 29.52 | Valeric acid | C_5_H_10_O_2_ | 102.068 | 103.075 | -2.8 | +H |
| 47 | 30.26 | 5-Hydroxy-3',6,7,4'-tetramethoxyflavone | C_19_H_18_O_7_ | 358.105 | 359.113 | 0.7 | +H |
| 48 | 30.74 | Caproicacidhexneylester | C_12_H_22_O_2_ | 198.162 | 199.170 | 1.1 | +H |
| 49 | 31.47 | Deoxy Arteannuin | C_15_H_20_O_2_ | 232.146 | 233.154 | -0.4 | +H |
| 50 | 31.54 | Methyl arteannuate | C_16_H_24_O_2_ | 248.178 | 249.185 | -0.5 | +H |
| 51 | 32.71 | (+)-Artemisinic acid | C_15_H_22_O_2_ | 234.162 | 235.169 | -0.2 | +H |
| 52 | 32.73 | Methyl 3-phenyl propenoate | C_10_H_10_O_2_ | 162.068 | 163.075 | -1.1 | +H |
| 53 | 33.51 | Quercetagetin-6,7,3',4'-tetramethylether | C_19_H_18_O_8_ | 374.100 | 397.091 | 4.1 | +Na |
| 54 | 35.35 | Arginine | C_6_H_14_N_4_O_2_ | 174.112 | 213.076 | 4.7 | +K |
| 55 | 37.57 | β-Sitosterol | C_29_H_50_O | 414.386 | 453.348 | -2.3 | +K |
| 56 | 40.62 | 1,2,3,5,6,8a-hexahydro-4,7-dimethyl-1-(1-methylethyl)naphthalene | C_15_H_24_ | 204.188 | 205.195 | 0.3 | +H |
| 57 | 40.62 | 2-Hexenal | C_6_H_10_O | 98.073 | 99.080 | -2.6 | +H |
| 58 | 40.62 | Cis-Carveol | C_10_H_16_O | 152.120 | 153.128 | 2.4 | +H |
